# Supplementary material for: Patterns of Intron Gain and Loss in Fungi
Source: PLoS Biol. 2004 Nov 30;2(12):e422. doi: 10.1371/journal.pbio.0020422 (PMC532390; doi:10.1371/journal.pbio.0020422)
Supplement: Table S1 — Also available at http://genes.mit.edu/NielsenEtAl/. (4.3 MB ZIP). [file pbio.0020422.st001.zip › NielsenEtAl/html/1234.html]

AN2967.1.NCU00950.1.MG01648.1.FG06863.1


```
 CLUSTAL W (1.82) Multiple Sequence Alignments - Introns Inserted


Sequence 1: MG01648.1	518 aa
Sequence 2: FG06863.1	416 aa
Sequence 3: AN2967.1	437 aa
Sequence 4: NCU00950.1	475 aa
Alignment Length: 544 aa
Number Identitical Residues: 176 aa
Alignment Score (without introns) 8664


MG01648.1 	MAGGFLSGICLRIAHLQAYTS0VQYLPK0IGLIGFSAVTATRGGSPAYI1NPSEHRKSRL
NCU00950.1	---------------------~------~--------------------~----------
FG06863.1 	---------------------~------~--------------------~----------
AN2967.1  	---------------------~------~--------------------~----------
          	                                                            

MG01648.1 	SNLQSLPNYVLEC2MHDKARQRTAK2QLGSRAMTSMAANTVPIPRRGVDYRGKIVLAPMV
NCU00950.1	----------MTS~VTEETTAPVLA~AAAAAAAAGLTQKRVPIPPRGVDYRGKIVLAPMV
FG06863.1 	-------------~-----------~-------MATEVKRVPIPRRGVDYRGKVVLAPMV
AN2967.1  	----------MTS~C----------~-----------PKRVPIPANGVDYRGKVVLAPMV
          	            .                         : **** .*******:******

MG01648.1 	RSSELPSRLMALHYGADLVWG1PETVDYSMIGTTRRLNPKTNVLQWTRLQNNGQKQAPPD
NCU00950.1	RSGELPSRLLALHYGADLVWG1PETIDRAMIGTTRRVNPRNHCIEWTRVPTNNVKKE--L
FG06863.1 	RSGELPSRLLALKYGADLVWG1PETVDYSMIGTSRRFDEESKTLEWFRTSSHGQKDPPPD
AN2967.1  	RSGELPSRLLALKYGADLCWG~PETIDRAMIGTTRRVNPRNGCIEFTRMPSNSVNKD--D
          	**.******:**:***** ** ***:* :****:**.: ..  ::: *  .:. :.    

MG01648.1 	AKENAMFTMHPELEGKRLIFQLGSGDPERAVAAARLVANDVAGIDLNAGCPKPFSTTGGM
NCU00950.1	FDESVIYRIDPVREAGRHIFQIGTSDPARAVECAKLVAADVSGIDVNAGCPKPFSTHAGM
FG06863.1 	AKESIIYRLHPELEKDKLIFQIGTADPDRAVEAARLVAADVAGIDVNAGCPKPFSTSGGM
AN2967.1  	AKESVIYRLDPAREKGKLIFQMGTANPELAVQAGKLVAADVNGIDVNSGCPKPFSTSGGM
          	 .*. :: :.*  *  : ***:*:.:*  ** ..:*** ** ***:*:******** .**

MG01648.1 	GAALLRDPDRLVAILEALVREVAPAAEIGISVKIRLLETAAETEALVRRLVKTGITALTV
NCU00950.1	GAALLRTPEKLVSILEALVAEIKPAFDIGISVKIRLLETPAETEALVRRLCATGITGLTI
FG06863.1 	GAALLQTPDKLCAILEALVKNITPEFEIGISVKIRLLDTAAETEALVRRLCATGITGLTI
AN2967.1  	GAALLRTPDKLVSILEALVKEVGEPYQIGISVKIRLLETPELTESLVSRLVRTGITGLTI
          	*****: *::* :****** ::    :**********:*.  **:** **  ****.**:

MG01648.1 	HCRTTPMRPRERAIRGQLRMVADVCREAGVACLMNGDVKDRDEGVRLAAEYGTDGAMIAR
NCU00950.1	HCRTTPMRPRERAIRGQLRMIADVCHEFGVACLVNGDVENRDHALELMAEYGTDGAMIAT
FG06863.1 	HCRTTPMRPRERAIRGQLRMIAEVCHEFGVACLVNGDVEGRDHGLQLAEEFGADGAMIAV
AN2967.1  	HCRTTPMRPRERAIRDQLRMIVSTCHNAGVACIMNGDVTSRDEALALMQEYGADGAMIAT
          	***************.****:...*:: ****::**** .**..: *  *:*:****** 

MG01648.1 	AAEENPSCFRAGADGGLAPWTETVPLFMRFCLDMENKFSNTKYLLNNMIPGKAPEFRPTQ
NCU00950.1	CAEKNGSCFRAKADGGLATWEEAVAKYVRFAMEVENKFHNTKFMLTQMVPGKSPKYRELT
FG06863.1 	AAEKNPSCFRSKANGGVAPWEEVVKQYVKTSIEVDNRFGNTKFLLSNMIPGKSKFYQPVN
AN2967.1  	AAEANSSCFRSEADGGLAPWREVVHEYVKLCIECENRFGNTKYLINMLIPGKDKEFREAK
          	.** * ****: *:**:*.* *.*  ::: .:: :*:* ***:::. ::***   ::   

MG01648.1 	HGKSYLALCEALN---LEDLVEQAKALDDKLGLTERQEKSKPVAPLEKKKKQKQNNPA--
NCU00950.1	PCKSYGKIIEVLGLTGDEELVEMARRTDELMKLVTPDGSSSNSSSSSGNDNSKVEAAGDD
FG06863.1 	QSKTYKSICEALC---LDDYLEAAIAVDKRRGLG---------QPLQGKAAKKAAAKA--
AN2967.1  	QAKCYTDLCTIL---QHDDLMPAAQEVDEILGIAHKSTARSKAVENAIANNDTARAAG--
          	  * *  :   *     :: :  *   *.   :   .   .          ..    .  

MG01648.1 	--------ALASGGNMSKARDA------SAKPRKPLSERGVGSRQEESHITKAAALEPAT
NCU00950.1	KKKHDLTNNVAQQENLKRKREDGDEETEKQQPPPSMRINVSEAGEAATTAATSVAEQQSS
FG06863.1 	--------LAAANAQSPKSEQG------KEQKRK---RKMSDSRPTKQVKASEPTPVAAT
AN2967.1  	--------GVSRSKNTSPATGGIGPIRTNSIPKPMKAETKAESKPAATDVDVSTPAPQPS
          	          :   :        .    .             :           .   .:

MG01648.1 	LNT-------
NCU00950.1	QPVAAAVAAS
FG06863.1 	L---------
AN2967.1  	QNEVAV----
          	    :
```
